# Effect of Partial Root Drying Stress on Improvement in Tomato Production

**DOI:** 10.3390/cimb47020084

**Published:** 2025-01-28

**Authors:** Huilian Xu, Hairong Jing, Runyu Shi, Minghao Chen, Chunfang Wang, Qicong Xu, Jianfang Bai, Xiaoyong Liu, Mengmeng Kong

**Affiliations:** 1 School of Biological Science and Technology, University of Jinan, Jinan 250024, China; 042172104438@ujn.edu.cn (H.X.); 202221100274@stu.ujn.edu.cn (H.J.); bio_shiry@stu.ujn.edu.cn (R.S.); bio_chenmh@stu.ujn.edu.cn (M.C.); bio_liuxy@ujn.edu.cn (X.L.); 2 Institute of Hydroecology, MWR & CAS, Wuhan 430079, China; cx251820@outlook.com; 3 International Nature Farming Research Center, Matsumoto 390-1401, Japan; qicongx@163.com; 4 Beijing Academy of Agriculture and Forestry Sciences, Beijing 100097, China; baijianfang131@163.com

**Keywords:** fruit yield, nitrate reductase activity, proline contents, antioxidant enzyme, nitrate reductase gene (*NR1*), drought stress genes (*DREB3*), tomato plants

## Abstract

Several countries around the world are facing the issue of freshwater availability, where agriculture is highly dependent on irrigation, consuming 70% of this vital resource. Water availability is the most limiting factor for the crop production sector and one of the main regulators of the spatial distribution of plants. It is noted that in recent years, the methods of irrigation water application have been improved. Currently, research is directed towards irrigation strategies that reduce water applications. A partial root drying (PRD) technique involves irrigating one-half of the root zone while leaving the other half in relatively dry soil. This method is used in the production of various crops, such as potatoes and cotton. However, the mechanism of PRD, including the physiological and molecular biological processes involved, is not fully understood. In this study, tomato plants were treated with PRD and nitrogen (N) top-dressing. The results showed that PRD could significantly increase the fruit yield, photosynthetic activities, nitrate reductase activity, and fruit quality in the tomato plants, and PRD could also promote the concentrations of oxygen species (O_2_^−^), malondialdehyde (MDA) and proline contents, and activities of antioxidant enzymes. In addition, PRD could enhance stress resistance by increasing disease resistance and NP1 and DRED3 antioxidant enzyme activity. Tomato plants treated with PRD compared to the control showed high photosynthetic activity, high yield, better quality of production, and low leaf blight incidence. Overall, the results indicate that PRD is a feasible approach that could be effectively utilized in tomato fields to improve plant growth and production compared with the control.

## 1. Introduction

The newly coined term “xerophytophysiology” describes the physical, mechanical, and biochemical mechanisms by which plants respond to drought-related factors, such as soil water deficiency, low humidity, salinity, and intense irradiation [1]. In the study of water stress and drought resistance in plants, passive measures—specifically, how to make plants resistant to water deficits and cultivate drought-resistant varieties—have always been a primary focus of research [2]. The mechanisms of plant xerophytophysiology can be actively or intentionally utilized in crop production to achieve desired results, including high-quality fruits and strong resistance to diseases, pests, and stresses [1]. In this context, “drought” is not always a true water stress that harms the plant; rather, it serves as a stimulus signal that promotes internal modifications beneficial to the plant, such as increased levels of anthocyanins including cyanidin-3-O-rutinoside, peonidin-3-O-rutinoside, and an unknown cyanidin pentaglycoside derivative [3]. When there is no actual drought, the treatment often acts as a false signal. The linked processes within the plant change in response to this signal as it is transmitted through the molecular regulation system [4].

The application of partial root drying (PRD) is an irrigation technique in which one-half of the root zone is irrigated while the other half is allowed to dry out; then, the well-irrigated side is alternately dried down [5]. Recently, PRD has been successfully applied to vegetable crops such as tomatoes [6,7], potatoes [4,8], rapeseed [9], peppers [10], and beans [11], as well as to fruit trees such as grapevines [12,13], pears [14], and apples [15]. PRD technology, developed based on root-to-shoot signal transduction, is one of the active applications of xerophytophysiology in plant production, especially in locations where drought is not necessarily present [1]. The irrigation method of partial root-zone drying (PRD) relies on drought stimulation without actual water stress in order to induce xerophytophysiological signaling and regulations [16].

Several studies have attempted to elucidate the molecular mechanisms involved [16,17,18]. When a plant perceives a drought stimulus, it transmits the signals to its internal genetic system, activating the relevant genes and leading to biochemical and physiological responses in reaction to the drought, regardless of whether this stimulus results in a real or perceived water deficit [16,17]. Many researchers attribute these benefits solely to non-hydraulic chemical signals, often overlooking the roles of internal physiological regulation and gene expression [19]. In the present study, the physiological regulation of tomato plants treated with PRD focuses on osmotic regulation, leaf turgor maintenance, water-use efficiency, and related physiological activities.

Excessive nitrogen fertilization can lead to the passivation of some active genes in crop seeds. Plants exhibit a form of intelligence that enables them to perceive changes in environmental conditions [20]. When a crisis stimulus occurs, it transmits signals to the DNA, prompting the activation of corresponding genes that initiate a series of reactions to resist the adverse environment [16]. Certain stimuli can activate previously inactive genes in plants, such as the nitrate reductase gene (*NR1*), and drought stress genes (*DREB3*) in tomato plants [21]. As a result, improved plant resistance, fruit quality, and levels of reactive oxygen species (O_2_^−^), malondialdehyde (MDA), antioxidant enzymes, and proline can be expected when exposed to drought factors, including soil water deficit, low humidity, salinity, and strong light exposure [22,23]. Studies have demonstrated that partial root drying, also referred to as alternate irrigation on both sides (PRD), significantly enhances fruit yield and quality by inducing osmotic adjustment, maintaining turgor potential, and supporting photosynthetic activities [1,16].

In short, tomato plants subjected to PRD receive environmental stimulation and undergo internal adaptive adjustments, including osmotic regulation. This process improves physiological activities and enhances resistance to both abiotic and biotic stresses [21]. In this study, we aimed to elucidate the mechanisms by which PRD benefits agronomic traits, physiological and biological activities, plant–water relations, and gene regulation. This study investigates PRD as a sustainable approach to tomato production. First, we evaluate the fruit yield, photosynthetic activities, nitrate reductase activity, and fruit quality in the tomato plants. Second, we characterize the concentrations of oxygen species (O_2_^−^), malondialdehyde (MDA), and proline contents, as well as the activities of antioxidant enzymes. Third, we assess stress resistance by increasing disease resistance and NP1 and DRED3 antioxidant enzyme activity. Overall, the results demonstrate that PRD is a feasible approach that can be utilized in tomato cultivation to enhance plant growth and production.

## 2. Materials and Methods

### 2.1. Plant Materials and Irrigation Treatments

#### 2.1.1. Greenhouse Experiment

Tomato plants were obtained by germinating tomato seeds and growing them under controlled conditions (25 °C, 16 h light/8 h dark, 60% relative humidity) for 20 days. Seedlings of a large fruit species of tomato (*Lycopersicon esculentum* Mill. Cv. Myoko) were transplanted into a natural soil-based polyethylene rainout greenhouse. The soil, classified as volcanic ash (Andosol), had the following Tadashi’s properties [24]. Based on dry mass, organic fertilizer was applied at an optimum rate of 200 g/m^2^, which was the optimum dose [25]. This organic fertilizer, made from a mixture of oil sludge (3 parts), rice bran (6 parts), and fish meal (1 part), was fermented with a microbial inoculant (*Lactobacillus* and yeast). The organic fertilizer contained total nitrogen, available phosphorus, and available potassium levels of 52 g kg^−2^, 30 g kg^−1^, and 20 g kg^−1^, respectively. Irrigation pipes, positioned 30 cm from the tomato plants, provided water. In the partial root drying (PRD) plots, one side of the row of plants was watered while the other side was allowed to dry. After two weeks, the previously dried side was irrigated, and the wet side was allowed to dry. This alternation of irrigation was conducted every two weeks. PRD was the designed experiment, and nitrogen top-dressing was the positive control. The four treatments were (1) control, (2) PRD, (3) N top-dressing, and (4) PRD + N top-dressing.

#### 2.1.2. Pot Experiment in the Incubator

**Figure 1:**
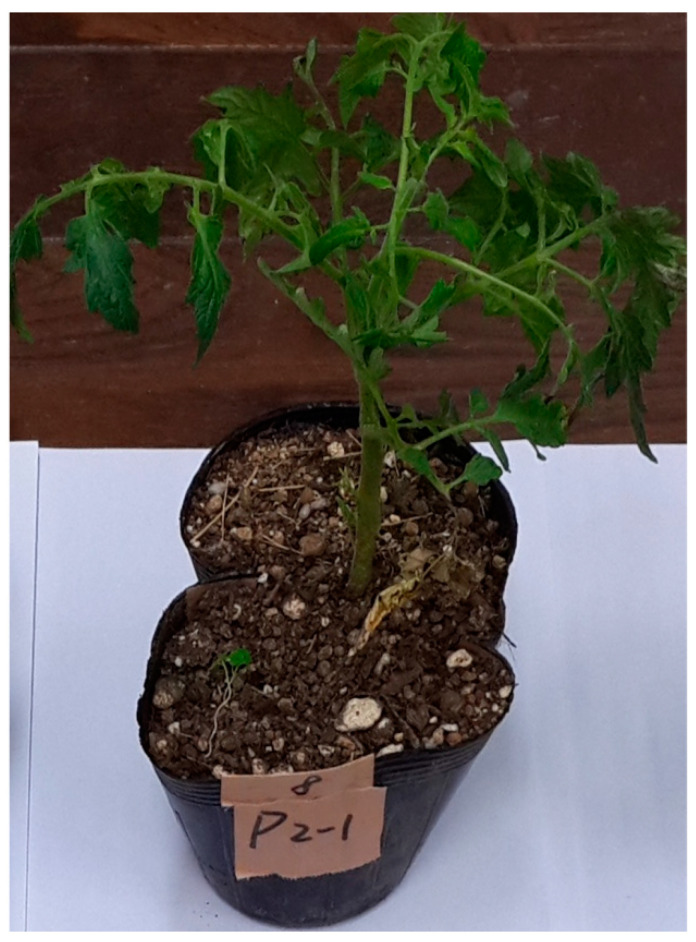
The tween pots for the partial root drying treatment in tomato plants.

The pot experiment utilized the same variety of tomato plants. As shown in Figure 1, two plastic pots (150 cm^3^ in volume and 11 cm in height) were connected with double-sided tape. Tomato seedlings were planted in the middle of the twin pot for 20 days, with the roots spreading into both sides of the twin pot. A unilateral root drying treatment (PRD) was applied for two weeks, followed by irrigation and subsequent alternate drying of the other side. The experiment was designed with or without PRD and with or without nitrogen top-dressing. A total of four treatments, each with 20 pots, were randomly placed on the shelf of the artificial growth chamber. The light intensity (PPF, photosynthetic photon flux) above the tomato canopy was maintained at 225 μmol m^−2^s^−1^.

#### 2.1.3. Measurement and Analysis of Photosynthesis

After the tomato plants finished flowering on the fifth truss in the greenhouse experiment, the fully expanded fifth truss was used to measure photosynthesis. The Li-6400 system (LI-COR Biosciences, Lincoln, NE, USA) was employed to assess the photosynthetic rate (PN) of the newly fully unfolded youngest leaves under different PPF conditions. The light response curve was expressed as PN = PC (1-e-KI)-RD, where PC represents the photosynthetic capacity, RD is the respiration rate, I is the PPF, K is a constant, and YQ is the maximum quantum yield, which is indicated by the initial slope of the curve and calculated using the formula YQ = KPC. The relative humidity in the leaf chamber was maintained at 60 ± 5%, fluctuating according to transpiration, while the air temperature was 21.5 °C, which also varied slightly with changes in light intensity.

#### 2.1.4. Analysis of Osmotic Adjustment and Leaf Turgor Maintenance

**Figure 2:**
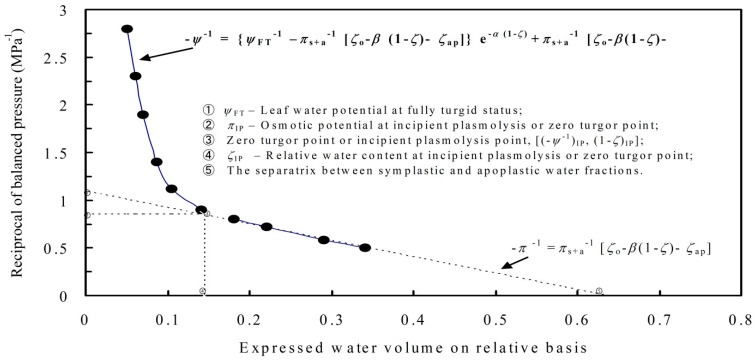
A schematic model of the pressure–volume curve.

As shown in Figure 2, Xu’s modified pressure–volume (P-V) curve equation (Xu et al., 2012) was used to analyze the osmotic adjustment, turgor maintenance, and cell water compartment of tomato leaves [21]. The P-V curve equation is −*Ψ*^−1^ = (*Ψ*_FT_^−1^ − *π*_s+a_^−1^(*ζ*_o_ − *β*(1 − *ζ*) − *ζ*_ap_))}e^−*α*(1−*ζ*)^ + *p*_s+a_^−1^(*ζ*_o_ −*β*(1 −*ζ*) − *ζ*_ap_), where *Ψ* is leaf water potential (MPa); *Ψ*_FT_ is *Ψ* at water saturation; *π*_s+a_ is the average osmotic potential in the symplast and apoplast; *ζ* is the leaf relative water content for a certain time point; ζ_o_ is ζ on the fully turgid status; *α* and *β* are related constants; *ζ*_ap_ as the proportion of *ζ* in the apoplast (cell walls); and 1 − *ζ*_ap_ is proportion of *ζ* in the symplast. For a cell, the physiological activities will be higher when the proportion of water in the symplasm is larger, where the most important biochemical reactions occur, or the proportion of water in the apoplast (cell wall) is smaller, where the most important chemical reactions do not occur. The P-V equation is modified from a minus exponential equation, y = Y_o_e^−αx^, where Y_o_ is the beginning maximum value of y. Originally, the equation was used to determine the absorption coefficient of colored liquid. If we let −*Ψ*^−1^ = y, *Ψ*_FT_^−1^ = *Y*_1_, *π*_s+a_^−1^ = *Y*_2_, 1−*ζ* = x, *ζ*_o_ = *x*_o_, and *ζ*_ap_ = *x*_b_, the equation can be simplified as y = (*Y*_1_ − *Y*_2_(*x*_o_ − *βx*−*x*_b_))*e*^−*α*x^ + *Y*_2_(x_o_− *βx*−*x*_b_); if the correction term, (x_o_ − *βx* −*x*_b_), is neglected, the equation will be y = (*Y*_1_ − *Y*_2_)*e*^−*α*x^ + *Y*_2_, which is a simple minus exponential curve. As shown in Figure 2, the less deeply sloped linear part of the curve was modeled by the following equation: −*p* ^−1^ = *p*_s+a_^−1^ [*z*_o_ − *b* (1 − *z*) − *z*_ap_]. In theory, when this linear curve meets the exponential curve, the leaf turgor potential reaches 0, where cell walls will separate from the cell membrane, i.e., the phenomenon of plasmolysis. This zero-turgor point is also called an incipient plasmolysis point. In many cases, the linear curve and the exponential curve reach closer and closer but do not cross until (1 − *z*) reaches a large value. Therefore, the incipient plasmolysis point is defined as the point [(−*y*^−1^)_IP_, (1 − *z*)_IP_], where (−*y*^−1^)_IP_ = (−*p*^−1^ + *d*) = (−*y*^−1^ − *d*) (*d* = 0.01((−*y*^−1^)_IP_)). In other words, when the distance between *f*(−*y*^−1^) and *f*(−*p*^−1^) reaches 2% of −*y*^−1^, i.e., 2*d*, the incipient plasmolysis point ((−*p*^−1^ + *d*) or (−*y*^−1^ − *d*)) is determined. The point where the curve of *f*(−*p*^−1^) crosses with the abscissa is the separatrix between the symplastic and apoplastic water fractions. The abbreviations of the above symbols and their units were shown in our previous study [1].

#### 2.1.5. Determination of Nitrate Reductase Activities

The NO_2_-produced NO_2_^−^ can be extracted by a phosphoric acid buffer solution containing cysteine and EDTA at pH 8.7. The concentration of NO_2_ in the reaction solution was measured and used to indicate the level of enzyme activity. The NO_2_ concentration was determined using the sulfonamide (sulfanilamide) colorimetric method, a conventionally sensitive technique [26].

#### 2.1.6. Determination of Oxidant Enzyme Activities

According to Beyer and Fridovich (1987) and Chakrabarty et al. (2009), the activity of superoxide dismutase (SOD) was determined using the riboflavin–nitroblue tetrazolium (NBT) method to detect O_2_^−^ concentration [27,28]. Catalase (CAT) activity was measured by assessing the elimination of H_2_O_2_ by spectrometry at 240 nm, according to Beers and Sizer (1952) [29]. POD activity was measured based on the methodology described by Khalil et al. (2006), using guaiacol as the oxidation substrate [30]. The concentration of malondialdehyde (MDA), a decomposition product of peroxidized polyunsaturated fatty acids in membrane lipids, was quantified by colorimetry using the thiobarbituric acid method [31]. The presence of the free superoxide radical (O_2_^−^) was assessed by monitoring the nitrite formation from hydroxylamine in the presence of O_2_, as per Bissenbaev et al. (2007) [32].

#### 2.1.7. Analysis of the Expression of the Nitrate Reductase (NR1) and Stress-Related (DREB3)

The expression of the NR1 gene was analyzed using real-time PCR after 12 h of different treatments. Total RNA was extracted from tomato leaf samples using the QIAGEN RNA Easy kit, and cDNA was synthesized from the total RNA with the PrimeScript II 1st strand cDNA synthesis kit. Primers for the *NR1* gene were designed based on its gene sequence registered in GenBank, utilizing Primer Premier 5 and Sigma Corporation. The forward primer was F-5′-GGTTGAGGTGCTTGACTT-3′, and the reverse primer was R-5′-CTCCCTTGTGAGGTTTGC-3′. The expression of the tomato stress resistance gene (DREB3) was also analyzed using real-time PCR. The primer sequences for DREB3 were F-5′-ATGATAATAATGTCTACAGAGCAA-3′ and R-5′-CTAATGTTGCCATAAAAAC TCTC-3′, with an amplified product length of 160 bp. Real-time PCR analysis was performed with cDNA as the template and the actingene (SlactinF-5′-GGAATGGGACAGAAGGAT-3′; SlactinR-5′-CAGTCAGGAACAGGGT-3′) as the internal control.

### 2.2. Statistical Analysis

All experiments were conducted using a completely randomized design. The experimental data were analyzed statistically using SPSS 25.0 (IBMCorp., Armonk, NY, USA). Means were separated using Tukey’s HSD test at *p* ≤ 0.05 following ANOVA.

## 3. Results

### 3.1. Identification of NR1 and DREB3 Gene

**Figure 3:**
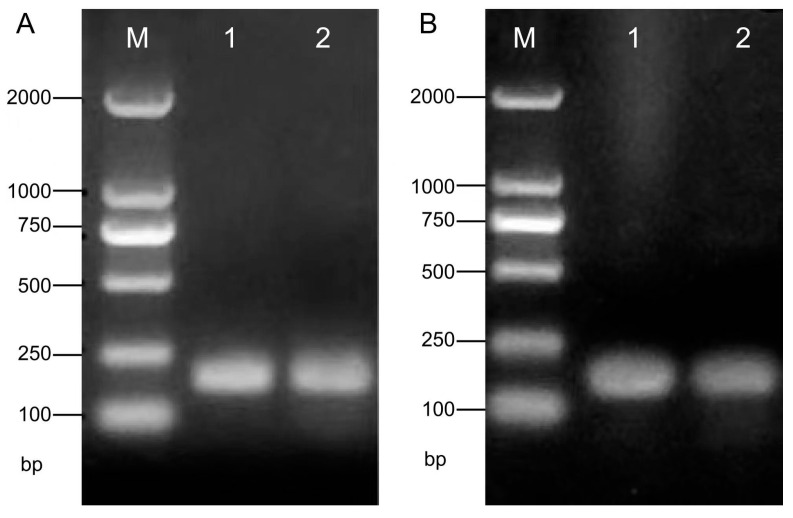
Detection of selected genes in tomato plants. PCR products of (**A**) nitrate re-educated (NR1) and (**B**) drought stress DREB3 genes were detected. M is the DL2000 marker; 1 and 2 are the PCR amplitude profiles of the sample.

The RT-PCR analysis was conducted to detect selected genes from tomato plants treated for PRD. The target fragment length for the *NR1* gene was 160 bp, and the *DREB3* gene also measured 160 bp, as confirmed by sequencing results (Figure 3). The PCR amplification results indicated that the selected genes, nitrate reductase *NR1* and drought resistance gene *DREB3*, produced strong bands in the gel, corresponding to the target cDNA fragments of the *NR1* and *DREB3* genes in the tomato plants (Figure 3A,B). The results demonstrated that the genes of *NR1* and *DREB3* could be up-transcribed at the cDNA level.

### 3.2. Fruit Yield

In the soil-based greenhouse experiment, the partial root drying (PRD) treatment increased fruit yield by 24.3% without nitrogen (N) top-dressing and by 12.2% with N top-dressing, compared to the control (Table 1). The improvements in fruit production due to PRD were attributed to increases in fruit size of 13.9% without top-dressing and 9% with N top-dressing (Table 1). Overall, nitrogen fertilizer top-dressing diminished the yield-enhancing effect of PRD.

Additionally, N top-dressing significantly increased fruit yield by 7.3% in the control plot and by 5.6% in the PRD-treated plot. These increases in fruit yield from N top-dressing were accompanied by improvements in fruit size of 6.4% in the control plot and 5.6% in the PRD plot. However, there were no increases in fruit numbers in either the control or PRD plots (Table 1). In conclusion, PRD increased tomato fruit yield regardless of N top-dressing, while N top-dressing only enhanced fruit yield in the absence of PRD treatment. Furthermore, negative interactions exist between PRD and N top-dressing concerning fruit production and yield components. In the pot culture, no fruit was harvested; however, both PRD and N top-dressing significantly increased biomass yield (*p* ≤ 0.05) (Table 1).

### 3.3. Photosynthetic Activities

#### 3.3.1. Soil-Based Experiment

The PRD treatment significantly increased photosynthetic capacity (*P_C_*) by 13.9% and 19.3% without and with top-dressing, respectively, as shown in Table 1. The effects of PRD on the respiration rate (*R_D_*) and on the maximum quantum yield (*Y_Q_*) were similar to those on *P_C_*. However, the effects of nitrogen (N) on *Y_Q_* were greater (9.5%) than those of PRD (14.3%). N top-dressing increased *P_C_* by 9.5% and 19.3% without and with PRD, respectively (Table 1). The effect of N on *R_D_* was larger in the PRD plot (7.2%) than in the control plot. The effect of N on *Y_Q_* was similar to that on *P_C_*. There were no interactive effects between PRD and N on *P_C_*, *R_D_*, or *Y_Q_* (Table 1).

#### 3.3.2. Pot Experiment

In the pot experiment, PRD significantly improved *P*_C_ and related *Y*_Q_ and *R*_D_, while N top-dressing did not affect photosynthetic activities. This finding contrasts with the results from the soil-based experiment (Table 1).

### 3.4. Effects on Nitrate Reductase Activity

#### 3.4.1. Soil-Based Experiment

At both the seedling and later fruit harvest stages, N top-dressing increased nitrate reductase activity, but PRD showed no effect. Soluble proteins indicate the total number of total enzymes, including nitrate reductase. Both PRD and N increased the concentration of soluble proteins (Table 1). There was no interaction between PRD and N top-dressing on nitrate reductase activity or the soluble protein concentration. Although PRD did not increase nitrate activity, it did increase soluble proteins (Table 1). This indicates that PRD may enhance the concentrations of other enzymes, which will be discussed in later paragraphs.

#### 3.4.2. Pot Experiment

In the pot experiment, the effects of PRD and N on nitrate reductase activity and soluble protein concentration exhibited similar trends to those in the soil-based experiment (Table 1). N top-dressing increased both nitrate reductase activity and soluble protein levels.

### 3.5. Fruit Quality

PRD slightly decreased calcium levels in fruit, potentially due to disturbances in leaf transpiration. The results indicated that PRD improved the fruit quality by increasing the concentrations of sugars, including glucose, organic acid, and vitamin C (Table 2). Conversely, top-dressing resulted in decreased levels of the fruit quality components, including sugars, organic acids, vitamin C, and calcium (*p* ≤ 0.05). There were no interactions observed between PRD and nitrogen on fruit quality components.

### 3.6. Concentrations of Oxygen Species (O_2_^−^) and Malondialdehyde (MDA) and Proline Contents

#### 3.6.1. Superoxide (O_2_^−^)

At the early growth (seedling stage) stage of the greenhouse-cultivated tomato crops, PRD significantly increased O_2_^−^ concentration in both plots by 21.4% and 37.4% with and without N top-dressing (Table 3). However, N top-dressing decreased the O_2_^−^ concentration by 30.0% without PRD. During the later growth (fruiting period) stage, the effects of PRD and N top-dressing were similar to those observed in the later fruit harvest stage. In the soil-based greenhouse experiment, the early growth stage corresponded to the completion of flowering for the first truss finished with fruit set up, and the later growth stage was when the fruit on the fifth truss was ripe. In the pot culture experiment, at both the early and later stages, the effects of PRD and N top-dressing were consistent with those observed in the soil-based greenhouse experiment.

#### 3.6.2. Malondialdehyde (MDA)

In the greenhouse experiment, N top-dressing decreased MDA by 3.8% with PRD and 11.5% without PRD in the early stage and by 1.8% with PRD and 3.0% without PRD in the later stage (Table 3). However, in the pot culture experiment, both PRD and N top-dressing had no effect on MDA in either the early or later stages.

#### 3.6.3. Proline

In the greenhouse experiment, PRD significantly increased the proline concentration by 28.4% with nitrogen (N) and by 34.1% without nitrogen during the early stage (Table 3). However, in the later stage, the effect of PRD diminished in the N top-dressing plot, while it exhibited a notable 200.0% increase in the plot without N top-dressing. This phenomenon may be attributed to the acclimation of proline-forming mechanisms to the PRD treatment. In addition, N top-dressing increased proline levels without PRD but decreased proline levels with PRD (Table 3). Thus, there were interactive effects between PRD and N top-dressing on the proline concentration. In the pot experiment, PRD consistently increased the proline concentration, both with and without N top-dressing, in both the early and later stages. However, N top-dressing had no effect on the proline levels in the pot culture. Under conditions of drought, saline–alkali stress, high temperature, low temperature, and freezing stress, the proline content increased significantly.

### 3.7. Activities of Antioxidant Enzymes

#### 3.7.1. Super Oxide Dismutase (SOD)

In the greenhouse experiment, PRD significantly increased SOD activity by 30.4% (with N) and 44.9% (without N) in the early stage and by 100.1% (with N) and 100.4% (without N) in the later stage. N top-dressing decreased SOD activity in both plants with and without PRD at both the early and later stages (Table 3). In the pot experiment, PRD similarly increased SOD activity with and without N top-dressing at both the early and later stages. As in the greenhouse experiment, N top-dressing decreased SOD activity in both plants with and without PRD at both the early and later stages.

#### 3.7.2. Peroxidase (POD)

In both greenhouse and pot experiments, PRD increased POD activity both with and without N top-dressing at the early and later stages. However, in all treatment plots, N top-dressing decreased POD activity. There was no interaction between PRD and N top-dressing (Table 3). Overall, the results indicated that in both the pot and greenhouse experiments, POD activity significantly increased with PRD, regardless of N top-dressing.

#### 3.7.3. Catalase (CAT)

In the greenhouse experiment, the results showed that PRD significantly increased CAT activity, both with and without N top-dressing, at both the early and later stages. During the early stage, N top-dressing significantly decreased CAT activity when used alone, but had little to no effect when combined with PRD, indicating an interaction between PRD and N top-dressing (Table 3). However, at the later stage, N top-dressing had no effect on CAT activity without PRD, but significantly decreased CAT activity when PRD was applied, indicating a different interaction than that observed in the early stage. In the pot experiment, neither PRD nor N top-dressing affected CAT activity during the early stage, but both had a significant impact at the later stage.

### 3.8. Analyses of the P-V Curve and Osmotic Adjustment

#### 3.8.1. Osmotic Adjustment

As shown in Table 4, the greenhouse experiment at the early stage revealed that the leaf osmotic potentials (*π*) at fully turgid status (*π*_FT_) and midday (*π*_MD_) were lower (more negative) in the PRD treatments compared to those without PRD. N top-dressing increased both *π*_FT_ and *π*_MD_ (making them less negative). Since leaf water potential (*Ψ*) at fully turgid status (π_FT_) and midday (*Ψ*_MD_) showed no differences among treatments, and turgor potential (*P*), is defined as the difference between *Ψ* and π (i.e., *P* = *Ψ*_−π_)*,* it follows that P was higher in the PRD treatments than in those without PRD. The decreases in *π* were attributed to increases in the osmolyte concentration within the cell. The osmotic adjustment refers to the additional active increases in osmolyte concentration (Δ*C*_osm_) beyond the original osmolyte concentration.

As shown in Table 5, Δ*C*_osm_ was higher in the PRD treatments than in the N treatments, while Δ*C*_osm_ was lower in the N top-dressing treatments. These increases in osmolyte concentration were supported by soluble sugars, which are components of the osmolytes. Similar results were observed in the pot experiment. Typically, osmotic adjustment leads to greater resistance to cell dehydration. *π*_IP_ is the osmotic potential at the time point when cell call walls begin to separate from the cell membrane, indicating incipient plasmolysis. A lower *π*_IP_ correlates with a higher dehydration resistance, and the same holds true for the leaf relative water content at incipient plasmolysis (*ζ*_IP_). Both PRD and N top-dressing treatments decreased *π*_IP_ and *ζ*_IP_. The results of the pot experiment show trends similar to those in the greenhouse experiment, eliminating the need for further detailed explanations.

#### 3.8.2. Cell Water Compartments

Cell water is stored in the symplast and apoplast (cell walls), referred to as the symplastic water fraction (*ζ*_sym_) and the apoplastic water fraction (*ζ*_apo_). At a relative level, *ζ*_sym_ is defined as *ζ*_sym_ = 1 − *ζ*_apo_. When osmotic concentration increases in the symplast, water from the apoplast moves across the membrane into the symplast, where more biochemical processes occur, making it crucial for the plant to withstand environmental stresses (Table 4). *ζ*_sym_ was higher in the PRD treatments compared to those without PRD, and it was greater in the N top-dressing treatments than in the control plots. The pot experiment yielded results similar to those observed in the greenhouse experiment.

### 3.9. Expressions of the Nitrate Reductase Gene and the Dehydration-Responsive Element-Binding Protein Gene

#### 3.9.1. Expression of the Nitrate Reductase Gene

Nitrate is the primary form of soil nitrogen available to plants. Nitrate reductase (EC 1.6.6.1) first reduces nitrate to nitrite before it can be incorporated into amino acids. Subsequently, nitrite reductase (EC 1.7.7.1) reduces nitrite to ammonium. Therefore, the function of nitrate reductase is to prevent the excessive accumulation of nitrate, which can be toxic in high amounts. Many internal and dehydration-responsive element-binding protein genes increased *NR1* expression in the greenhouse experiment with nitrogen (N) top-dressing, particularly at the later stages, as shown in Table 5. N top-dressing significantly increased *NR1* expression both with and without partial root-zone drying (PRD) at both early and later stages. In the pot experiment, PRD had no effect on *NR1* expression in the early stage but increased *NR1* expression in the later stage. N top-dressing dramatically elevated *NR1* expression at both the early and later stages. These results confirm that nitrate is the most critical factor in inducing *NR1* gene expression.

#### 3.9.2. Expressions of the Dehydration-Responsive Element-Binding Protein Gene

In the greenhouse and pot conditions, at both the early and later stages, the dehydration-responsive element-binding protein gene (*DREB3*) was significantly up-regulated by PRD treatments in both plots, regardless of N top-dressing. There were no significant interactions between PRD and N top-dressing (see Table 5). In the pot culture, *DREB3* was significantly up-regulated by PRD, particularly at the later stage. Additionally, there were significant interactions between PRD and N top-dressing, especially during the later stage, indicating that N top-dressing diminished the effect of PRD at this time.

## 4. Discussion

Water stress and drought resistance in plants are passive measures that have always been a primary focus of research—specifically, research aims to determine how to make plants resistant to water deficits and cultivate drought-resistant varieties. The mechanisms of plant xerophytophysiology can be actively or intentionally utilized in crop production to achieve desired results, including high-quality fruits and strong resistance to diseases, pests, and stresses [1,2]. In this experiment, tomato crops significantly benefited from PRD treatment in terms of yield and fruit quality. The higher leaf water potential observed at midday indicated that the plants were not experiencing water stress, despite being subjected to PRD treatments (Table 1). It is reasonable to expect that increasing fruit size while reducing fruit number can compensate for yield loss. PRD reduced the boll number of cotton plants but did not cause an increase in boll size to offset the loss of fruit [33]. In the soil-based greenhouse experiment, the treatment of partial root drying (PRD) increased fruit yield by 24.3% without N top-dressing and 12.2% with N top-dressing as compared to the control (Table 1). Responses to partial root drying (PRD) may vary depending on plant genotype and the degree or duration of drying [34]. Liu et al. (2006) reported that PRD significantly increased ABA concentration in the xylem sap of potato plants, but the midday leaf water potential was similar to that of fully irrigated plants [8]. Dembinska et al. (1992) studied split roots using PRD and found that changes in ABA concentration did not reduce leaf water potential [35]. In cereals, pollen sterility caused by different ABA treatments on stems and leaves is comparable to that resulting from low leaf water potential [36].

This discrepancy may arise from differences between cotton and tomato crops or from variations in soil types, such as the cracked clay soils of Australia versus the organic-rich volcanic ash (Andosol) used in this study. As a plant production technique, partial root-zone drying (PRD) has been implemented in numerous crops and fruit trees [10,12,37,38]. However, the underlying mechanisms, especially those related to the osmotic adjustment and cell water compartments, remain unclear. PRD acts as a deliberate stimulus that encourages healthier plant growth by inducing a series of xerophytic physiological regulations [1]. It does not induce actual water stress but triggers signals that mimic real water stress. At the molecular level, plants can sense changes in environmental conditions—such as drought induced by high UV radiation, soil water deficit, low humidity, rhizosphere salinity, extreme temperatures, and pests—and transmit signals internally to DNA to induce the expression of related genes, activate metabolism related to physiological and morphological regulation, and enhance stress tolerance [39]. The PCR amplification result showed that the selected genes of nitrate reductase *NR1* and drought resistance gene *DREB3* were detected to obtain the strong bands identified in the gel target cDNA fragments of the *NR1* gene and *DREB3* in tomato plants (Figure 3A,B). The results demonstrated that the *NR1* and *DREB3* genes could be up-transcribed at the cDNA level (Figure 3) and also showed that PRD improved the fruit quality by increasing the concentrations of sugars, including glucose, sucrose, organic acid, and vitamin C (Table 2).

Under drought conditions, the commonly observed responses include plant hormone regulation, osmotic adjustment, and morphological strengthening, all of which are based on xerophytophysiological mechanisms [1]. Scientists have traditionally taken a passive approach, focusing on breeding varieties that exhibit high resistance to adverse environmental conditions. However, this resilience can be actively induced through the mechanisms of drought physiology. In the present study, mild or false drought stimulation was applied to tomato plants, resulting in healthier and stronger plants. PRD increased the cell–water ratio in the symplasm (*ζ*_sym_). The leaf osmotic potential (π_FT_) was highly treated with PRD (Table 4), leading to a higher leaf turgor potential compared to the control. This change was attributed to a greater active accumulation of solutes (∆*C*_osm_) (Table 5). The improved leaf turgor potential and a higher symplastic water ratio through osmotic adjustment may explain the enhanced physiological activity observed in PRD-treated plants. Water in the symplast is directly related to biochemical metabolisms, whereas water in the apoplast (cell wall) is not directly involved in biochemical processes within the cytoplasm [2]. Therefore, we posit that a higher water content in the symplasm is more favorable for biochemical metabolism, with plant tissues having a higher symplastic water ratio exhibiting greater physiological activity [40]. The most important physiological adaptation to drought stimulation is osmotic adjustment. The active accumulation of solutes lowers the osmotic potential, resulting in a higher turgor potential at a given water potential level. Turgor potential serves as the driving force for cell growth and stomatal opening.

Superoxide (O_2_^−^) is a type of free radical. Although excessive accumulation of O_2_^−^ can be toxic to plant cells, it plays a crucial role in plant disease resistance and response to stress [9]. At the early growth (seedling stage) stage of the greenhouse-cultivated tomato crops, PRD significantly increased the O_2_^−^ concentration in both plots by 21.4% and 37.4% with and without N top-dressing (Table 3). However, N top-dressing decreased the O_2_^−^ concentration by 30.0% without PRD. At the later growth stage, the effects of PRD and N top-dressing were similar to those observed during the fruit harvest stage. Malondialdehyde (MDA) is a common index for measuring the degree of oxidative stress, reflecting membrane lipid peroxidation in plants. In the soil-based greenhouse experiment, N top-dressing decreased MDA levels by 3.8% with PRD and 11.5% without PRD during the early stage and by 1.8% with PRD and 3.0% without PRD in the later stage. However, in the pot culture experiment, neither PRD nor N top-dressing had any effect on MDA levels at either stage (Table 3). Proline, a component of proteins, is widely present in its free state in plants [1]. In the greenhouse experiment, PRD dramatically increased proline concentration by 28.4% with N top-dressing and 34.1% without N top-dressing during the early stage (Table 3). However, in the later stage, this effect disappeared in the N top-dressing plot, although a 200.0% increase was observed in the plot without N top-dressing. In the pot experiment, PRD increased proline levels both with and without N top-dressing during both the early and later stages. Proline content in plants reflects their stress resistance to some extent; varieties with strong drought resistance exhibit greater proline accumulation [41].

Superoxide dismutase (SOD) is an antioxidant enzyme in organisms [21]. In the greenhouse experiment, PRD significantly increased SOD activity by 30.4% with N top-dressing and 44.9% without N top-dressing in the early stage and by 100.1% with N top-dressing and 100.4% without N top-dressing in the later stage. N top-dressing decreased SOD activity in both plants with and without PRD during both stages. Similarly, in the pot experiment, PRD increased SOD activity with and without N top-dressing at both the early and later stages. In both experiments, N top-dressing decreased POD activity across all treatment plots, with no interaction between PRD and N top-dressing. POD serves multiple functions, including eliminating the toxicity of hydrogen peroxide, phenols, amines, aldehydes, and benzene. Plants possess several classes of PODs, with up to 160 enzymes found in cereal plants. In the greenhouse experiment, PRD increased CAT activity with and without N top-dressing during both the early and later stages. In the pot experiment, both PRD and N top-dressing showed no effect on CAT in the early stage but significantly impacted CAT activity in the later stage. It has been reported that, compared to SOD and POD, CAT only shows its responses when stress is applied to a significant extent or for an extended period (Table 3).

The plant leaves treated with PRD exhibited a higher solute concentration, a larger symplastic water ratio, lower osmotic potential, and relative water content at incipient plasmolysis, resulting in increased stress resistance in PRD-treated plants. The enhancement in stress resistance was reflected in the improved activity of several antioxidant enzymes, such as NR1 and DREB3. The photosynthetic activity of tomato leaves in PRD plots was also higher, correlating with improved leaf turgor potential. Our results indicate that PRD-induced osmotic adjustment improved photosynthesis, increased disease resistance, and enhanced antioxidant enzyme activity by regulating the expression of the *NR1* and *DERB3* genes. Furthermore, nitrogen top-dressing induced the activity of nitrate reductase and weakened the impact of PRD (Table 5). Tomato plants treated with PRD demonstrated high photosynthetic activity, high yield, good fruit quality, and low leaf blight incidence. Overall, the results suggest that PRD is a feasible approach that could be utilized in tomato fields to enhance plant growth and production.

## 5. Conclusions

Tomato plants were treated with PRD and N top-dressing. The results showed that PRD significantly induced osmotic regulation, maintaining leaf turgor potential above normal levels. Additionally, it enhanced the resistance of tomato plants to leaf blight by increasing the solute concentration, symplast water ratio, osmotic potential, and relative water content at incipient plasmolysis. These factors indicate greater stress resistance in PRD-treated plants.

PRD enhances stress resistance through osmotic adjustment, improved photosynthesis, increased disease resistance, and elevated activities of the antioxidant enzymes NP1 and DRED3. Tomato plants treated with PRD exhibited high photosynthetic activity, increased yield, and better production quality, along with reduced leaf blight incidence. Overall, the results demonstrate that PRD is a viable approach for improving plant growth and production in tomato fields.

## Figures and Tables

**Table 1:** Fruit yield (greenhouse test) or biomass yield (pot experiment), photosynthetic activity (*P*_C_), maximum quantum yield (*Y*_Q_), dark respiration rate (*R*_D_), soluble proteins (g kg^−1^), and nitrate reductase activity affected by PRD and N top-dressing.

Treatment	Fruit (g pl^−1^)	*P*_C_ Size (g)	*R*_D_ (μmoL m^−2^ s^−1^)	*Y*_Q_ (moL moL^−1^)	Soluble Proteins Early (g kg^−1^)	Nitrogen Reductase	
						Later (g kg^−1^)	Early (g kg^−1^)
Zoilo seabed rainout greenhouse							
PRD + N	3502 ***	105.8 **	27.7 **	2.7 **	0.0659 **	6.74 **	7.17 ***
PRD	3406 **	101.0 **	24.3 *	2.4 *	0.0570 *	5.96 *	6.79 **
N	3120 *	97.1 *	25.2 *	2.3 *	0.0597 *	6.18 *	6.36 *
control	2741	88.7	22.8	2.1	0.0537	5.64	5.94
pot experiment							
PRD + N	0.342 *	-	17.9 **	1.8 ***	0.0415 *	5.06 **	5.41 ***
PRD	0.321	-	17.2 **	1.7 **	0.0422 *	4.08 *	4.62 *
N	0.326	-	16.3 *	1.6 *	0.0361	4.98 **	4.91 **
Control	0.304	-	15.2	1.4	0.0374	4.29	4.21

Note: “ *, **, ***” show statistical significance.

**Table 2:** Concentrations of sugars (sucrose, glucose, and fructose), organic acid (OA), vitamin C (Vc), and calcium affected by PRD and N top-dressing.

Treatments	Glu	Fru	Sugars	OA	V_C_	Ca
	kg^−1^					
PRD + N	5.1 **	35.4 **	31.6 **	72.1 **	10.7 **	0.379 **
PRD	5.7 ***	40.0 ***	35.7 ***	81.5 ***	12.1 ***	0.432 ***
N	4.2 *	27.3 *	23.8 *	55.2 *	8.4 *	0.319 *
control	4.8	31.7	27.8	64.3	9.0	0.359

Note: “*, **, ***” show statistical significance.

**Table 3:** Concentrations of oxygen species (O_2_^−^), malondialdehyde (MDA), and proline, as well as activities of antioxidant enzymes in tomato leaves at early and later stages, as affected by PRD and N top-dressing.

Treatment	O_2_	MDA		Proline		SOD		POD		CAT	
PRD N	(μmol/g)	(μmol/g)		(μmol/g)		(μmol/g)		(μmol/g)		(μmol/g)	
	Later	Early	Later	Early	Later	Early	Later	Early	Later	Early	Later
Zoilo seabed rainout greenhouse											
PRD + N	2.27 **	2.01 **	4.26 *	4.75 **	4.8 *	5.28 **	870 **	733 **	3819 **	4255 **	1.36 **
PRD	2.57 ***	2.29 ***	4.38 **	4.96 ***	13.9 **	5.87 ***	967 ***	815 ***	4246 ***	4730 ***	1.45 ***
N	1.29 *	1.85 *	4.21 *	4.44 *	6.69 *	3.64 *	373 *	603 *	3528 *	2948 *	1.07 *
control	1.87	2.09	4.34	3.70	4.57	4.05	403	692	3923	3278	1.27
pot experiment											
PRD + N	1.57 **	1.86	8.49	8.57 *	7.37 *	6.31 ***	328 **	481 **	2856 **	2008	2.733 *
PRD	1.98 ***	1.82	8.43	8.25 *	6.92 *	3.53 **	493 ***	638 ***	3092 ***	2375 *	2.987 **
N	1.06 *	1.86	8.60	7.47	3.25	2.77 *	191 *	426 *	2308 *	1908	0.875
control	1.31	1.81	8.53	7.64	3.48	2.87	265	475	2600	2133	0.900

Note: “*, **, ***” show statistical significance.

**Table 4:** P-V curve analysis for parameters related to the osmotic adjustment of tomato leaves in response to partial root drying stimulation, *Ψ*, π and *P* leaf water potential, osmotic potential, etc., and turgor potential; ζ is leaf relative water content. The subscripts FT, MD, s + a, IP, and sym refer to fully turgid, midday, symplasm + apoplasm, incipient plasmolysis, and symplasm, respectively.

Treatment	*π*_FT_	*P*_FT_	*Ψ*_MD_	*π*_MD_	*P*_MD_	*π*_s+a_	*π*_IP_	*ζ*_IP_	*ζ*_sym_
Zoilo seabed rainout greenhouse (MPa)									
PRD + N	−0.239	−1.011	0.772	−0.964 *	−1.493 *	0.529 **	−0.841	−1.278 *	0.784 *
PRD	−0.241 *	−1.084 *	0.796	0.843 **	−0.957	−1.563	0.606 **	−0.864	−1.004
N	−0.223	−0.901	0.678	−0.975 *	−1.241 *	0.2666 *	−0.739 *	−1.229 *	0.839 *
control	−0.239	−0.932	0.694	0.693	−0.958	−1.316	0.358	−0.769	−1.236
pot experiment									
PRD + N	−0.224	−0.869 *	0.645 *	−0.711	−1.191 *	0.481 *	−0.811 *	−1.209 *	0.896 *
PRD	−0.221	−0.873 *	0.652 *	−0.703	−1.186 *	0.483 *	−0.821 *	−1.212 *	0.895 *
N	−0.206	−0.769	0.563	−0.693 *	−1.052	0.359	−0.701	−1.117	0.883
control	−0.207	−0.753	0.546	−0.701	−1.074	0.373	−0.719	−1.125	0.882

Note: “*, **” show statistical significance.

**Table 5:** The increase in osmotic concentration (Δ*C*_osm_) and soluble sugar concentration, as well as expressions of *NR1* and *DREB3* genes.

Treatment	Δ*C*_osm_	Soluble Sugars (g kg^−1^)		Nitrate		*NR1* Expression		*DREB3* Expression	
	(osmol m^−3^)	Early	Later	Early	Later	Early	Later	Early	Later
Zoilo seabed rainout greenhouse									
PRD + N	414.5 **	45.1 **	530 **	418 **	-	1.70	2.44 **	2.09 *	2.03 **
PRD	444.4 **	75.0 ***	599 ***	473 ***	-	1.11 *	2.16 **	2.81 **	2.58 ***
N	369.4 *	0.0 *	446 *	335 *	-	0.86**	0.83 *	1.52	1.22 *
control	382.1	12.7	495	371	-	0.76	0.36	1.73	1.68
pot experiment									
PRD + N	356.3 *	47.6 **	499 **	406 **	-	1.57	4.32 **	1.48 *	1.85 *
PRD	357.9 *	49.2 **	423 *	322 *	-	1.00 *	1.89 *	1.86 **	4.33 ***
N	308.7	0.0 *	423 *	322 *	-	1.53 *	1.51 *	0.98	0.85 **
control	315.3	6.6	330	271	-	0.91	0.27	1.00	1.33

Note: “*, **, ***” show statistical significance.

## Data Availability

The original contributions presented in this study are included in this article. Further inquiries can be directed to the corresponding authors.

## References

[1] Xu H.L., Bai J.F., Kawabata S., Chang T.T. (2023). Applications of Xerophytophysiology and Signal Transduction in Plant Production—Flower Qualities in *Eustoma grandiflorum* Were Improved by Sub-Irrigation. Sustainability.

[2] Düring H., Dry P.R., Botting D.G., Loveys B. (1996). Effects of partial root-zone drying on grapevine vigor, yield, the composition of fruit and use of water. Proceedings of the Ninth Australian Wine Industry Technical Conference.

[3] Xiang M.Q., Ding W.S., Wu C., Wang W.J., Ye S.W., Cai C.Y., Hu X., Wang N.N., Bai W.Y., Tang X.S. (2021). Production of purple Ma bamboo (*Dendrocalamus latiflorus Munro*) with enhanced drought and cold stress tolerance by engineering anthocyanin biosynthesis. Planta.

[4] Kang G.Z., Li G.Z., Xu W., Peng X.Q., Han Q.X., Zhu Y.J., Guo T.C. (2012). Proteomics reveals the effects of salicylic acid on growth and tolerance to subsequent drought stress in wheat. J. Proteome Res..

[5] Stoll M., Loveys B., Dry P. (2000). Improving water use efficiency of irrigated horticultural crops. J. Exp. Bot..

[6] Savic S., Stikic R., Srdic M., Savic D., Jovanovic Z., Prokic L.J., Zdravkovic J. (2004). The effect of partial root drying on growth and ions content and distribution on tomato. Acta Hortic..

[7] Mingo D.M., Bacon M.A., Davies W.J. (2003). Non-hydraulic regulation of fruit growth in tomato plants *(Lycopersicon esculentum* cv. Solairo) growing in drying soil. J. Exp. Bot..

[8] Liu F., Shahnazari A., Andersen M.N., Jacobsen S.-E., Jensen C.R. (2006). Effects of deficit irrigation (DI) and partial root drying (PRD) on gas exchange, biomass partitioning, and water use efficiency in potato. Sci. Hortic..

[9] Wang L., de Kroon H., Smits A.J.M. (2007). Combined effects of partial root drying and patchy fertilizer placement on nutrient acquisition and growth of oilseed rape. Plant Soil..

[10] Shao G.-C., Zhang Z.-Y., Liu N., Yu S.-E., Xing W.-G. (2008). Comparative effects of deficit irrigation (DI) and partial rootzone drying (PRD) on soil water distribution, water use, growth and yield in greenhouse grown hot pepper. Sci. Hortic..

[11] Wakrim R., Wahbi S., Tahi H., Aganchich B., Serraj R. (2005). Comparative effects of partial root drying (PRD) and regulated deficit irrigation (RDI) on water relations and water use efficiency in common bean (*Phaseolus vulgaris* L.). Agric. Ecosyst. Environ..

[12] Marsal J., Mata M., Del Campo J., Arbones A., Vallverdú X., Girona J., Olivo N. (2008). Evaluation of partial root-zone drying for potential field use as a deficit irrigation technique in commercial vineyards according to two different pipeline layouts. Irrig. Sci..

[13] Souza C.R.d., Maroco J.P., Chaves M.M., Santos T., Rodriguez A.S., Lopes C., Rodrigues M.L., Pereira J.S. (2004). Effects of partial root drying on the physiology and production of grapevines. Acta Horticulturae..

[14] Kang S., Hu X., Goodwin I., Jerie P. (2002). Soil water distribution, water use, and yield response to partial root zone drying under a shallow groundwater table condition in a pear orchard. Sci. Hortic..

[15] Leib B.G., Caspari H.W., Redulla C.A., Andrews P.K., Jabro J.J. (2006). Partial rootzone drying and deficit irrigation of ‘Fuji’apples in a semi-arid climate. Irrig. Sci..

[16] Su F.F., Li Y., Liu S.W., Liu Z.Y., Nie S.J., Xu Q.C., Qin F.F., Li F.L., Lyu D.Q., Xu H.L. (2020). Application of Xerophytophysiology and Signal Transduction in Plant Production: Partial Root-Zone Drying in Potato Crops. Potato Res..

[17] Hassan A., Amjad S.F., Saleem M.H., Yasmin H., Imran M., Riaz M., Ali Q., Joyia F.A., Ahmed S., Ali S. (2021). Foliar application of ascorbic acid enhances salinity stress tolerance in barley (*Hordeum vulgare* L.) through modulation of morpho-physio-biochemical attributes, ions uptake, osmo-protectants and stress response genes expression. Saudi J. Biol. Sci..

[18] Davies F.S., Davies F.S., Albrigo L.G. (1994). Citrus 2 (Crop Production Science in Horticulture).

[19] Gowing D.J.G., Davies W.J., Jones H.G. (1990). A positive root-sourced signal as an indicator of soil drying in apple, Malus x domestica Borkh. J. Exp. Bot..

[20] Xu H.L., Xu Q.C., Li F.L., Feng Y.Z., Qin F.F., Fang W. (2012). Applications of xerophytophysiology in plant production—LED blue light as a stimulus improved the tomato crop. Sci. Hortic..

[21] Chattha M.S., Ali Q., Haroon M., Afzal M.J., Javed T., Hussain S., Mahmood T., Solanki M.K., Umar A., Abbas W. (2022). Enhancement of nitrogen use efficiency through agronomic and molecular based approaches in cotton. Front. Plant Sci..

[22] Manghwar H., Hussain A., Ali Q., Saleem M.H., Abualreesh M.H., Alatawi A., Ali S., Munis M.F.H. (2021). Disease Severity, Resistance Analysis, and Expression Profiling of Pathogenesis-Related Protein Genes after the Inoculation of *Fusarium equiseti* in Wheat. Agronomy.

[23] Ali S., Shakoor A., AliI Q., Chattha M.S., EL-SHEIKH M.A., Ali S. (2021). Oxidative Stress Alleviation as Indicated by Enzymatic and Nonenzymatic Antioxidants and Osmoregulators in Barley (Hordeum vulgare L.) under Salt (Nacl) Stress by Ascorbic Acid (AsA). Pak. J. Bot..

[24] Tadashi T., Sadao S. (2002). Distribution and Classification of Volcanic Ash Soils. Glob. Environ. Res. Engl. Ed..

[25] Hanxi W., Jianling X., Xuejun L., Di Z., Longwei L., Wei L., Lianxi S. (2019). Effects of long-term application of organic fertilizer on improving organic matter content and retarding acidity in red soil from China. Soil Tillage Res..

[26] Sophia A.E., Abdellatif A.L., Laila I., Caterina M., Salvatore C., Aziz A. (2017). A sensitive method for the determination of Sulfonamides in seawater samples by Solid Phase Extraction and UV–Visible spectrophotometry. Spectrochim. Acta Part A Mol. Biomol. Spectrosc..

[27] Beyer W.F., Fridovich I. (1987). Assaying for superoxide dismutase activity: Some large consequences of minor changes in conditions. Anal. Biochem..

[28] Chakrabarty D., Verma A.K., Datta S.K. (2009). Oxidative stress and antioxidant activity as the basis of senescence in Hemerocallis (day lily) flowers. J. Hortic. For..

[29] Beers R.F., Sizer I.W. (1952). Aspectrophotometric method for measuring the breakdown of hydrogen peroxide by catalase. J. Biol. Chem..

[30] Khalil N.M., Mello M.A.M., França S.C., Oliveira L.A.A., Oliveira O.M.M.F. (2006). Callus cell culture of *Pothomorphe umbellata* (L.) under stress condition leads to high content of peroxidase enzyme. Eclética Química..

[31] Dey S.K., Dey J., Patra S., Pothal D. (2007). Changes in the antioxidative enzyme activities and lipid peroxidation in wheat seedlings exposed to cadmium and lead stress. Brazilian J. Plant Physiol..

[32] Bissenbaev A.K., Altybaeva N.A., Kolbaeva G.A. (2007). Role of reactive oxygen species and antioxidant enzymes in hormone regulating programmed cell death of wheat aleurone layer. J. Cell Mol. Biol..

[33] Neilsen J., Constable G. (2006). Investigation into Partial Root Zone Drying in Cotton Cropping Systems.

[34] Xu H.L., Qin F.F., Xu Q.C., Tan J.Y., Liu G.M. (2011). Applications of xerophytophysiology in plant production–The potato crop improved by partial root zone drying of early season but not whole season. Sci. Hortic..

[35] Dembinska O., Lalonde S., Saini H.S. (1992). Evidence against the regulation of grain set by spikelet abscisic acid levels in water-stressed wheat. Plant Physiol..

[36] Saini H.S., Westgate M.E. (1999). Reproductive development in grain crops during drought. Adv. Agron..

[37] Aganchich B., El Antari A., Wahbi S., Tahi H., Wakrim R., Serraj R. (2008). Fruit and oil quality of mature olive trees under partial rootzone drying. Grasasy Aceites..

[38] Spreer W., Ongprasert S., Hegele M., Wünsche J.N., Müller J. (2009). Yield and fruit development in mango (Mangifera indica L. cv. Chok Anan) under different irrigation regimes. Agric. Water Manag..

[39] Mulligan R.M., Chory J., Ecker J.R. (1997). Signaling in plants. Proc. Natl. Acad. Sci. USA.

[40] Patakas A., Noitsakis B. (1997). Cell wall elasticity as a mechanism to maintain favorable water relations during leaf ontogeny in grapevines. Am. J. Enol. Vitic..

[41] Wan L., Li X., Shi Y., He F., Jia Y. (2010). A study on the response and on the comparison of physiological and biochemicalindexes of four Lolium perenne varieties under PEG stress. Acta Prataculturae Sin..

